# *Bartonella rochalimae* in Raccoons, Coyotes, and Red Foxes

**DOI:** 10.3201/eid1512.081692

**Published:** 2009-12

**Authors:** Jennifer B. Henn, Bruno B. Chomel, Henri-Jean Boulouis, Rickie W. Kasten, William J. Murray, Gila K. Bar-Gal, Roni King, Jean-François Courreau, Gad Baneth

**Affiliations:** Napa County Health and Human Services, Napa, California, USA (J.B. Henn); University of California, Davis, California, USA (B.B. Chomel, R.W. Kasten); École Nationale Vétérinaire d’Alfort, Maisons-Alfort, France (H.-J. Boulouis, J.-F. Courreau); San José State University, San José, California, USA (W.J. Murray); Hebrew University of Jerusalem, Rehovot, Israel (G.K. Bar-Gal, G. Baneth); Nature Parks Authority, Jerusalem, Israel (R. King)

**Keywords:** *Bartonella rochalimae*, raccoon, red fox, coyote, bacteria, dispatch

## Abstract

To determine additional reservoirs for *Bartonella rochalimae*, we examined samples from several wildlife species. We isolated *B. rochalimae* from 1 red fox near Paris, France, and from 11 raccoons and 2 coyotes from California, USA. Co-infection with *B. vinsonii* subsp. *berkhoffii* was documented in 1 of the coyotes.

Twelve *Bartonella* species/subspecies have been recognized as zoonotic agents (*1*,*2*), including *B. rochalimae* isolated from a woman who had traveled from the United States to Peru before becoming ill with fever, splenomegaly, mild anemia, and rash (*3*). *B*. *rochalimae*, previously described as a *B. clarridgeiae*–like organism, has also been isolated from rural domestic dogs and gray foxes (*Urocyon cinereoargenteus*) from northern California (*4*,*5*). A case of fatal endocarditis in a domestic dog was associated with a *B. clarridgeiae*–like strain (*6*), later determined to be identical to *B. rochalimae* (5). Recently, *B. rochalimae* DNA was detected in a dog from Greece (*7*). High (43%) prevalence of bacteremia observed in gray foxes in California suggests that they might act as a wildlife reservoir for this newly identified species. Furthermore, several *B. clarridgeiae*–like and *B. rochalimae* genes have been detected in fleas collected from humans (*8*), rodents (*9*,*10*), red foxes (*Vulpes vulpes*) (*11*), and the environment in the Democratic Republic of Congo (*12*) during a plague outbreak. To determine whether other wildlife reservoirs exist, we tested samples from 3 additional wildlife species: coyotes, raccoons, and red foxes.

## The Study

From 1996 through 1999 in central coastal California, 21 *Canis latrans* coyotes (3 juveniles [<1 year of age] and 18 adults) and 42 *Procyon lotor* raccoons (11 juveniles and 31 adults) were trapped. In 2002, a blood sample was collected from a road-killed red fox near Paris, France. All samples were collected in EDTA tubes and frozen at −70°C until plated on heart infusion agar containing 5% rabbit blood and incubated in 5% CO_2_ at 35°C for up to 4 weeks (*13*); subsequently, extracted DNA was tested for *Bartonella* spp. by PCR. In addition, from May 2003 through September 2004, blood was collected from 42 red foxes (23 females and 19 males; 2 kits [<1 year] and 40 adults) in Israel, and extracted DNA was tested for *Bartonella* spp. by PCR.

*Bartonella* isolates from 2 (9.5%) coyotes (coyote 004 [7-month-old male] and coyote 22 [adult female captured in central California], which yielded 2 different-size colonies: coyote 22/sub1, large size; coyote 22/sub2, small size), 11 (26%) of the raccoons (7 adult females and 4 adult males), the 1 (100%) red fox from France, and DNA from the blood of 2 (5%) foxes from Israel were compared with *B. rochalimae* strains isolated from a human, rural dogs, and gray foxes. *Bartonella* isolates were analyzed by PCR restriction fragment length polymorphism (RFLP) of the 16S–23S intergenic transcribed spacer (ITS) region (all strains) and the *glt*A, *rpo*B and *fts*Z genes (raccoons, gray foxes, coyotes, and dogs), as previously described (*5*). For the isolate from the red fox from France, extracted DNA was also amplified for fragments of the *groEL* gene by using the primer sets HSPps1, HSPps2, and HSPsp4 (*11*,*14*). Sequencing was done in both directions by using a fluorescence-based automated sequencing system (Davis Sequencing, Davis, CA, USA). Sequences were imported into Vector NTI Suite 9.0 software (Invitrogen, Carlsbad, CA, USA) to obtain a consensus sequence. Align X in Vector NTI was used for aligning sequence variants with each other and other known *Bartonella* spp. for each of the 4 genes. A neighbor-joining tree was constructed in MEGA version 3.0 (www.megasoftware.net) by concatenating the 4 sequences. Bootstrap replicates were performed to estimate node reliability of the phylogenetic tree; values were obtained from 1,000 randomly selected samples of the aligned sequence data. Sequence data for the *groEL* gene of the isolate from the fox in France (GenBank accession no. FJ545656**)** was compared with sequences of DNA extracted from fleas collected on 4 foxes from Hungary (*11*) and deposited in GenBank under accession no. DQ522300.

**Figure 1:**
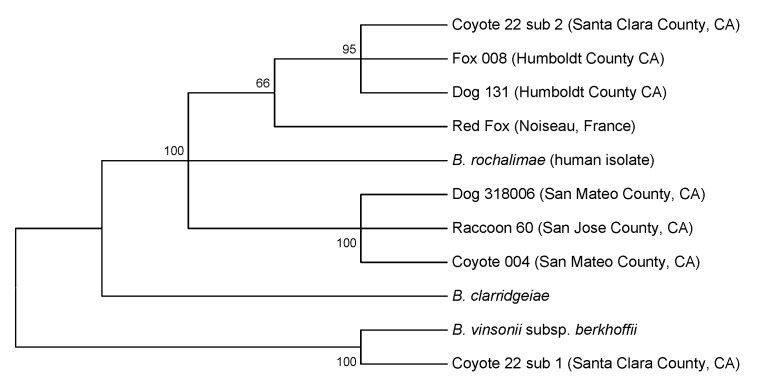
Phylogenetic tree of *Bartonella* species based on the combined *glt*A, *rpo*B, *fts*Z, and intergenic transcribed spacer sequence alignment. The tree shown is a neighbor-joining tree based on the Kimura two-parameter model of nucleotide substitution. Bootstrap values are based on 1,000 replicates. The analysis provided tree topology only; the lengths of the vertical and horizontal lines are not significant.

Amplified PCR products were obtained from the ITS region and the *glt*A, *rpo*B, and *fts*Z genes of all isolates. Isolates from coyote 004 and coyote 22/sub2, the red fox from France, and the 11 raccoons had identical RFLP profiles, also identical to those observed in the rural dogs and gray foxes from California (*5*). Isolate sub1 from coyote 22 had banding patterns identical to those of *Bartonella vinsonii* subsp. *berkhoffii* (American Type Culture Collection 51672), indicating co-infection with *B. vinsonii* subsp. *berkhoffii* and *B. rochalimae*. Consensus sequences were obtained for isolates from all coyotes and the red fox from France. Because the RFLP profiles for the 11 raccoon isolates were identical, only 2 isolates (from raccoons 60 and 75, adult females from central California) were selected for sequencing and were identical for all genes. Partial sequences from the 4 genes were identical for the isolates from coyote 004 and the 2 raccoons. These isolates were 100% similar to a strain recovered from the dog with endocarditis (GenBank accession nos. DQ676488–DQ676491) (*5*,*6*). The *B. rochalimae* isolate sub2 from coyote 22 was identical to isolates from rural dogs and gray foxes from northern California (accession nos. DQ676484–DQ676487). Similarity of isolates from these regions ranged from 99.5% to 100% (Tables 1, 2). A tree constructed from the merged set of concatenated sequences (Figure 1) demonstrates that isolate sub2 from coyote 22 clustered with isolates from the dog and gray fox from northern California; those from coyote 004 and raccoon 60 grouped with those from the dog with endocarditis.

**Table 1:** Percent similarity based on comparisons of the combined *glt*A, *rpo*B, *fts*Z, from 7 *Bartonella rochalimae* isolates

Isolate source	Red fox, France	Coyote 004	Coyote 22/sub2	Raccoon 60	Dog 318006	Dogs/gray foxes	Human
Red fox (Paris suburb, France)	100	99.5	99.8	99.6	99.5	99.9	99.8
Coyote 004 (San Mateo County, CA, USA)		100	99.6	100	100	99.6	99.6
Coyote 22/sub2 (Santa Clara County, CA, USA)			100	99.6	99.6	100	99.8
Raccoon 60 (San Jose, Santa Clara County, CA, USA)				100	100	99.6	99.6
Dog 318006 (San Mateo County, CA, USA)					100	99.6	99.6
Dogs/gray foxes (Humboldt County, CA, USA)						100	99.8
Human (Peru)							100

**Table 2:** Percent similarity based on comparisons of the intergenic transcribed spacer sequence alignment from 7 *Bartonella rochalimae* isolates

Isolate source	Red fox, France	Coyote 004	Coyote 22 sub2	Raccoon 60	Dog 318006	Dogs/gray foxes	Human
Red fox (Paris suburb, France)	100	99.5	99.8	99.6	99.5	99.9	99.8
Coyote 004 (San Mateo County, CA, USA)		100	99.6	100	100	99.6	99.6
Coyote 22/sub2 (Santa Clara County, CA, USA)			100	99.6	99.6	100	99.8
Raccoon 60 (San Jose, Santa Clara County, CA, USA)				100	100	99.6	99.6
Dog 318006 (San Mateo County, CA, USA)					100	99.6	99.6
Dogs/gray foxes (Humboldt County, CA, USA)						100	99.8
Human (Peru)							100

**Figure 2:**
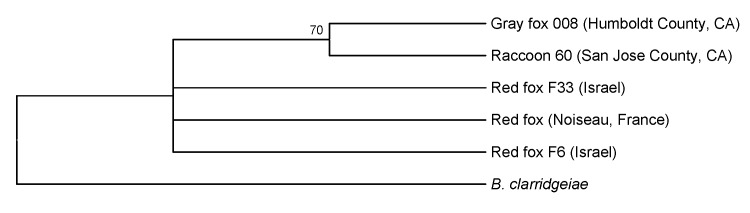
Phylogenetic tree of *Bartonella* species based on intergenic transcribed spacer sequence alignment for the isolates from the gray foxes, red foxes, and raccoons. Raccoon and gray fox isolates are shown for comparison. The tree shown is a neighbor-joining tree based on the Kimura 2-parameter model of nucleotide substitution. Bootstrap values are based on 1,000 replicates. The analysis provided tree topology only; the lengths of the vertical and horizontal lines are not significant.

According to PCR results and comparison of a 571-bp sequence amplified from the ITS region, the sequences from 2 foxes (1 male, 1 female) from 2 villages in northern Israel were identical to each other and to that from the fox from France (Figure 2). Differences of 2–5 bp were observed among ITS region sequences when comparing those from the foxes from Israel and France with those from *B. rochalimae* from gray foxes and raccoons from California. When the *groEL* partial sequence FJ545656 from the red fox from France was compared with sequence DQ522300 from a *Pulex irritans* flea collected from foxes from Hungary, the 156-bp fragment (based on the consensus sequence from both directions) from the red fox from France was 100% identical to that of the flea.

## Conclusions

We report the isolation or detection of *B. rochalimae* from red foxes, raccoons, and coyotes from North America, Europe, and the Middle East. Sequence analysis of 4 genes identified small variations in the *B. rochalimae* isolates from these different geographic regions. A relatively high percentage (26%) of raccoons had *B. rochalimae* bacteremia compared with only 9.5% (2/21) coyotes. A previous study found that of 109 coyotes, none were infected with *B. rochalimae*, but 31 (28%) harbored *B. vinsonii* subsp. *berkhoffii* (*13*). In raccoons, bacteremia was found in adults only, which is surprising because for all animals in general, *Bartonella* spp. bacteremia is detected more frequently in younger animals (*1*). Gray foxes from northern California had *B. rochalimae* bacteremia prevalence of 42% (*4*), suggesting that gray foxes and raccoons could be natural reservoirs of *B. rochalimae* in California and that infection of coyotes and domestic dogs could result from occasional spillover. Co-infection of a coyote also illustrates that wild canids can simultaneously harbor >1 species of *Bartonella*. Co-infection of humans with *B. henselae* and *B. vinsonii* subsp. *berkhoffii* has also been reported (*15*). Additionally, co-infection with 2 zoonotic *Bartonella* species in this coyote raises the possibility that humans and domestic dogs could also be co-infected with these species, making appropriate diagnosis more difficult. *Pulex* fleas collected on red foxes from Hungary (*11*) were indeed infected with a strain of *Bartonella* that was identical, at least for the *groEL* partial sequence, with that of the isolate from the red fox from France, suggesting that red foxes from central Europe may also be infected with *B. rochalimae*. Future studies with larger sample sizes will be needed to better define the role of these wild carnivores—red foxes, raccoons, and coyotes—in maintaining *B. rochalimae* in the environment.
